# Intra-Observer Reproducibility of Endoscopic Ultrasound Point Shear-Wave Elastography: A 120-Patient Prospective Cohort Study

**DOI:** 10.3390/medicina62040780

**Published:** 2026-04-17

**Authors:** Adrian Burdan, Bogdan Miutescu, Eyad Gadour, Calin Burciu, Mirela Danila, Felix Bende, Moga Tudor, Aymen Almuhaidb, Raluca Lupusoru, Andreea Brasovan, Roxana Sirli, Alina Popescu

**Affiliations:** 1 Advanced Regional Research Center in Gastroenterology and Hepatology, “Victor Babes” University of Medicine and Pharmacy, 300041 Timisoara, Romania; ghita-adrian.burdan@umft.ro (A.B.); danila.mirela@umft.ro (M.D.); bende.felix@umft.ro (F.B.); moga.tudor@umft.ro (M.T.); raluca.lupusoru@umft.ro (R.L.); andreea.brasovan@umft.ro (A.B.); sirli.roxana@umft.ro (R.S.); popescu.alina@umft.ro (A.P.); 2 Doctoral School, “Victor Babes” University of Medicine and Pharmacy, Eftimie Murgu Square 2, 300041 Timisoara, Romania; 3 Division of Gastroenterology and Hepatology, Department of Internal Medicine II, “Victor Babes” University of Medicine and Pharmacy, 300041 Timisoara, Romania; 4 Multiorgan Transplant Centre of Excellence, Liver Transplantation Unit, King Fahad Specialist Hospital, Dammam 32252, Saudi Arabia; dreyadgadour@gmail.com; 5 Department of Surgery, Imam Abdulrahman Bin Faisal University, Dammam 31441, Saudi Arabia; 6 Department of Gastroenterology, Western University of Medicine and Pharmacy “Vasile Goldis” of Arad, 317046 Arad, Romania; calin.burciu@umft.ro; 7 Department of Medicine, Gastroenterology Division, King Faisal Specialist Hospital and Research Center, Riyadh 11211, Saudi Arabia; draymen86.mu@gmail.com

**Keywords:** endoscopic ultrasound, shear-wave elastography, liver stiffness, reproducibility, EUS-pSWE

## Abstract

*Background and Objectives*: Endoscopic ultrasound point shear-wave elastography (EUS-pSWE) bypasses subcutaneous fat and may provide weight-independent liver stiffness measurements; however, data on reproducibility and quality criteria remain limited. This study aimed to evaluate the intra-observer reproducibility and short-term variability of EUS-pSWE. *Materials and Methods*: In this single-center prospective cohort study (December 2024–February 2025), 120 consecutive adults undergoing diagnostic EUS were enrolled. For each hepatic lobe, 10 consecutive measurements were obtained and grouped into two sequential blocks of five measurements without scope repositioning. Intra-observer reproducibility was assessed using intraclass correlation coefficients (ICC3,1). The agreement between acquisition runs and determinants of short-term variability was also evaluated. Same-day vibration-controlled transient elastography (VCTE) served as an external comparator. *Results*: Forty-six participants were obese (BMI ≥ 30 kg/m^2^). The mean VCTE stiffness was 6.24 kPa, while the mean EUS-pSWE stiffness was 9.40 ± 5.64 kPa. Among examinations meeting IQR/Median < 30% quality criteria, reproducibility was excellent (left ICC 0.97 [0.95–0.98]; right ICC 0.92 [0.86–0.95]) and consistent across BMI strata. EUS-pSWE correlated strongly with VCTE (r = 0.81, *p* < 0.001). In contrast, agreement between consecutive acquisition runs was low, indicating increased short-term variability. EUS-pSWE quality pass rates based on IQR/Median criteria were modest (left 56.7%, right 41.7%, both lobes 23.3%), although all measurements fulfilled device-specific validity criteria (VSN > 60%). Age and BMI were not significant predictors of variability. *Conclusions*: EUS-pSWE demonstrates excellent intra-observer reproducibility under quality-controlled conditions and shows a strong correlation with VCTE. However, short-term variability between acquisition runs and limited feasibility based on conventional quality thresholds should be considered. EUS-pSWE appears to be a promising modality for liver stiffness assessment, warranting further validation of quality criteria and clinical thresholds.

## 1. Introduction

Chronic liver disease (CLD) is now recognized as the *fourth* leading cause of years of life lost in people aged 30–59 years and claims almost two million lives every year—about 4% of all global deaths [1]. The overwhelming driver of this upward trend is metabolic dysfunction-associated steatotic liver disease (MASLD), the newly accepted umbrella term that replaces NAFLD and explicitly integrates metabolic risk factors [2,3]. Recent NHANES data comprising 13538 U.S. adults show that one-quarter of the population (25.6%) already fulfills MASLD criteria, with controlled-attenuation-parameter-defined steatosis present in 28.7% and stiffness-defined fibrosis in 11.3% [4]. Whole-population modeling predicts that, without effective interventions, MASLD prevalence will exceed 55% in adults by 2040, accompanied by a doubling of cirrhosis-related mortality [4].

Against this backdrop, non-invasive tests (NITs) such as vibration-controlled transient elastography (VCTE; FibroScan^®^) have become first-line tools for fibrosis staging because they are rapid, painless, and widely validated [5]. However, VCTE is technically unsuccessful in up to 18% of examinations, rising to 25–30% in patients with a body mass index (BMI) > 35 kg m^−2^ or in the presence of ascites; even when measurements are obtained, the diagnostic accuracy drops by ~30% in severe obesity [6,7,8]. Large prospective series confirm that female gender, age > 50 years, BMI > 30 kg m^−2^, and limited operator experience are independent predictors of unreliable or failed VCTE, underscoring an urgent need for complementary approaches in this expanding patient subgroup [8].

Endoscopic ultrasound point shear-wave elastography (EUS-pSWE) positions the ultrasound source within the gastric or duodenal wall, thereby bypassing subcutaneous fat and interposed bowel gas. Pilot studies report area-under-the-curve values between 0.86 and 0.92 for significant fibrosis—comparable to VCTE—while simultaneously allowing for full diagnostic EUS [9,10,11,12]. A recent prospective cohort of 112 patients demonstrated a sensitivity of 0.88 for ≥F2 fibrosis and showed that stiffness values obtained by EUS-SWE correlated strongly (r = 0.79) with histology [10]. However, robust data on reproducibility are still limited: only two small single-center series have published intra-observer intraclass correlation coefficients (ICCs), both reporting values in the 0.75–0.83 range but with wide confidence intervals and heterogeneous patient spectra [11,12].

The 2024 multiparametric ultrasound guideline from the World Federation for Ultrasound in Medicine and Biology (WFUMB) stipulates that an ICC ≥ 0.80, evaluated under real-world conditions, is mandatory before any elastography modality can be considered to be reliable for longitudinal monitoring or therapeutic decision-making [13]. The consensus document also emphasizes the importance of defining quality-control criteria (e.g., inter-quartile-range/median ratio < 0.30) and establishing modality-specific learning curves.

Potential cofounding factors of elastography precision include anthropometrics (BMI, waist circumference), liver-disease etiology (viral vs. metabolic vs. cholestatic), hepatic steatosis, and operator experience; however, none of these factors have been examined systematically for EUS-pSWE. For transabdominal devices, reliability improves after ~50 supervised examinations and plateaus after ~150, but the analogous learning curve data for the endoscopic ultrasound approach are currently limited and remain an area requiring further investigation [14,15]. Moreover, emerging evidence suggests that metabolic cofactors such as central obesity, diabetes, and sarcopenic obesity can increase within-observer variability by up to 25% [16,17,18]. Understanding how these variables interact with EUS-pSWE performance is crucial before widespread clinical adoption.

We therefore undertook a prospective cohort study to: (i) quantify intra-observer reproducibility of EUS-pSWE in a real-world MASLD-dominant population; (ii) benchmark its stiffness estimates against same-day VCTE measurements; and (iii) model the influence of BMI and underlying etiology on the measurement quality and failure rates [19,20].

## 2. Materials and Methods

### 2.1. Study Design and Population

We conducted a prospective, single-center cohort study between December 2024 and February 2025 at the Centre for Advanced Research in Gastroenterology and Hepatology, “Victor Babeș” University of Medicine and Pharmacy, Timisoara (Romania). The study protocol was approved by the Ethics Committee of Emergency County Hospital of Timisoara (No. 511 of 26 November 2024) and was prospectively registered on the Open Science Framework (OSF) registry (DOI: https://doi.org/10.17605/OSF.IO/3729P), ensuring the transparency and reproducibility of the study protocol. Consecutive adults (≥18 years) referred for clinically indicated endoscopic ultrasound (EUS) were screened in chronological order and enrolled after written informed consent, in accordance with the Declaration of Helsinki. EUS examinations were performed for standard clinical indications, including the evaluation of pancreatic lesions, biliary pathology, and subepithelial lesions. The exclusion criteria were: (i) focal hepatic lesion ≥ 1 cm; (ii) biliary obstruction (due to its known impact on liver stiffness measurements); (iii) ALT or AST elevation exceeding three times the upper limit of normal (ULN); (iv) right-sided heart failure or significant tricuspid regurgitation; (v) pregnancy or lactation; (vi) prior upper gastrointestinal surgery preventing standard scope positioning; (vii) inability to complete both FibroScan^®^ (Echosens, Paris, France) and EUS-pSWE during a single visit; and (viii) contraindications to deep propofol sedation.

For an anticipated ICC of 0.90 and a desired 95% CI width of ±0.06, the Zou formula yields *n* = 94. To allow for incomplete traces (~20% based on pilot audits), we targeted ≥120 participants, which preserves 80% power (β = 0.20) to confirm ICC > 0.80 at α = 0.05, even if quality failures occurred.

### 2.2. Elastography Protocols

All examinations were performed with an Olympus UCT-180 linear echo-endoscope (Olympus Medical Systems Corp., Tokyo, Japan) coupled to a Hitachi Arietta 850 ultrasound (FUJIFILM Healthcare, Tokyo, Japan) engine running the Point-ShearWave-Measurement firmware (ver 2.3.1). Patients fasted for ≥6 h and received monitored anesthesia care with propofol. Standard B-mode mapping identified vascular landmarks; Doppler was used to avoid vessels before placing shear-wave regions of interest (ROI).

In each hepatic lobe, 10 valid measurements were obtained: the left lobe from the gastric lesser curvature and the right lobe from the duodenal bulb. An oblong ROI (5 × 10 mm, depth > 10 mm) was positioned perpendicular to the capsule, avoiding large ducts or vessels.

A measurement was considered valid when the shear-wave propagation trace was stable and free of artifacts, with the system’s built-in quality indicators—the IQR/Median ratio and Velocity Signal-to-Noise (VSN)—monitored in real time. VSN was used as a device-specific quality indicator, and all measurements were acquired with VSN > 60%. These criteria define the measurement-level validity. For study analyses, additional quality criteria based on IQR/Median < 30% were applied at the lobe level to ensure comparability with established elastography standards.

Each hepatic lobe received 10 consecutive measurements, grouped into two sequential blocks of five measurements (initial and repeat) without scope repositioning. For analytical purposes, three complementary approaches were applied: (i) intra-acquisition reproducibility, assessed by comparing the two blocks of five measurements within each lobe; (ii) run-level repeatability, assessed by comparing summary measures (median of five measurements) between the two consecutive blocks; and (iii) reliability improvement, assessed by comparing single measurements versus aggregated measurements (median of five) across runs.

VCTE was performed prior to EUS by an experienced technician. The endosonographer performing EUS-pSWE was blinded to the VCTE results. The M-probe was used unless BMI ≥ 30 kg m^−2^, whereupon the XL-probe was selected automatically. Ten valid measurements with IQR/Median < 30% were mandated; the median value (kPa) was stored as FS MED and served both as an external comparator and for pathophysiology analyses.

Height and weight were measured on calibrated devices on the morning of endoscopy, and body mass index (BMI) was subsequently calculated. Laboratory tests within 48 h were abstracted from the electronic medical record. The liver disease etiology was assessed by the operator and coded as follows: 0 = none; 1 = MASLD; 2 = ALD; and 3 = other (viral, autoimmune, or cholestatic).

All EUS-pSWE measurements were performed by a single experienced endosonographer with extensive experience in diagnostic EUS. The operator had prior experience with EUS-based elastography techniques before study initiation.

### 2.3. Statistical Analysis

All analyses leveraged SPSS v27.0 (IBM, Armonk, NY, USA) and R 4.3.2 with the psych, irr and BlandAltmanLeh packages. Normality was tested via Shapiro–Wilk; continuous data are mean ± SD or median (IQR), as appropriate. Group comparisons used Welch t or Mann–Whitney U (two groups) and Kruskal–Wallis with Dunn–Bonferroni (≥3 groups). Categorical variables employed χ^2^ or Fisher exact. Intra-observer reliability ICC(3,1) was computed for each lobe with 95% CI; coefficients of variation (CoV) and Bland–Altman plots examined absolute agreement. The prespecified success criterion was ICC > 0.80 in the whole cohort and in BMI-stratified sub-groups.

Correlation and agreement between modalities were assessed as follows. Pearson’s r quantified the association between VCTE and EUS-pSWE stiffness values, while proportional bias was evaluated using Passing–Bablok regression. Logistic regression was used to test predictors of trace invalidity (outcome defined as IQR/Median ≥ 30%). Multivariate modeling was performed to identify the determinants of short-term measurement variability, defined as the absolute run-to-run difference in stiffness (ΔkPa). Variables with univariate *p* < 0.10 were entered into a linear regression model. Variance-inflation factor > 3 was used to assess multicollinearity, and model fit was expressed as adjusted R^2^.

Two-tailed *p* < 0.05 denoted statistical significance. Multiple testing was controlled using the Benjamini–Hochberg procedure (false-discovery rate 5%) for secondary analyses. Missing data (<2% of fields) were handled by pairwise deletion, as Little’s MCAR test confirmed randomness (χ^2^ = 12.4, *p* = 0.41).

### 2.4. Use of AI-Assisted Tools

AI-assisted tools were used for reference formatting and adaptation to the journal style.

## 3. Results

The final analytic cohort comprised 46 obese (BMI ≥ 30 kg/m^2^) and 74 non-obese participants. Obese patients were more frequently male than non-obese participants (58.7% vs. 30.2%, *p* = 0.008). Hypertension was more common in obese participants (63.0% vs. 39.0%, *p* = 0.043), while diabetes rates were numerically higher but did not differ significantly between groups (21.7% vs. 7.3%, *p* = 0.075). The serum ALT levels did not differ significantly between groups (33.2 ± 18.1 vs. 43.4 ± 38.9 U/L; *p* = 0.189) (Table 1).

The mean VCTE stiffness was similar in non-obese and obese participants (6.27 ± 4.04 vs. 6.20 ± 4.30 kPa; *p* = 0.929). Mean EUS-pSWE overall stiffness (mean of 10 consecutive measurements across both lobes, reported descriptively irrespective of lobe-specific quality criteria) was also comparable between BMI strata (9.05 ± 5.05 vs. 9.98 ± 6.50 kPa; *p* = 0.411). As expected for a surface-based technique, VCTE validity (IQR/Median < 30%) was numerically lower in obesity (93.5%) than in non-obesity (98.6%), though this difference did not reach statistical significance (*p* = 0.157).

In contrast, EUS-pSWE quality pass rates based on the IQR/Median < 30% criteria were lower, particularly for the right lobe (left lobe 56.7%, right lobe 41.7%; both lobes 23.3%). Importantly, all measurements fulfilled device-specific validity criteria (VSN > 60%), as described in the Methods section. The EUS-pSWE quality did not differ significantly with obesity. This likely reflects the application of strict IQR/median-based quality thresholds, which are well established for transabdominal elastography but not yet validated for EUS-pSWE. Therefore, the observed feasibility rates should be interpreted with caution, as they may underestimate the practical applicability of EUS-pSWE in routine clinical settings (Table 2).

Intra-observer reproducibility was evaluated by comparing the two consecutive blocks of five measurements (initial vs. repeat) within each lobe. Among examinations meeting the prespecified lobe-specific quality criterion (IQR/Median < 30%), reproducibility was excellent in both lobes (Table 3), and remained high in both BMI categories.

In a separate analysis, we evaluated the agreement between the two consecutive acquisition blocks (run 1 vs. run 2), each summarized by the median of five measurements. Unlike the high intra-acquisition ICCs reported above, the agreement between the run-level summary measures across the entire cohort was low (ICC 0.014 for the left lobe and 0.001 for the right lobe).

When stratified by BMI, non-obese patients retained moderate between-run reliability (left ICC 0.69; right ICC 0.64), whereas obese participants demonstrated markedly reduced repeatability (ICC ≤ 0.006). These findings suggest that short-term session-level variability may be more pronounced than measurement-level variability, particularly in obesity (Table 4).

The reliability improved when using the median of five measurements per run compared with using a single shot from each run. This pattern was observed in both lobes and across BMI categories (Table 5).

**Figure 1:**
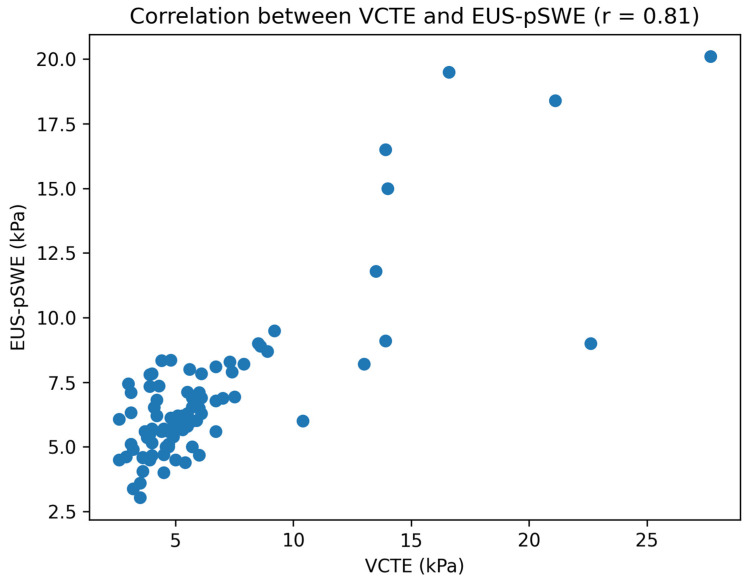
Scatter plot showing the correlation between VCTE and EUS-pSWE liver stiffness measurements. The solid line represents the linear regression fit (r = 0.81, *p* < 0.001).

EUS-pSWE and VCTE were strongly correlated overall (r = 0.81, *p* < 0.001), as illustrated in Figure 1, with similarly strong correlations in non-obese and obese participants (Table 6). Lobar EUS stiffness correlated strongly with VCTE, and the left and right lobe stiffness values were also strongly correlated. The correlation strength was comparable between lobes (r = 0.74 for the left lobe vs. r = 0.73 for the right lobe), suggesting that there was no clinically meaningful difference in performance between hepatic lobes.

In a multivariable linear model evaluating determinants of the absolute run-to-run difference (ΔkPa), neither age nor BMI was significantly associated with the measurement variability (Table 7), and the model explained little variance.

## 4. Discussion

### 4.1. Analysis of Findings

In this prospective cohort, EUS-pSWE demonstrated excellent intra-observer reproducibility when strict quality criteria were met, with ICC(3,1) values of 0.97 in the left lobe and 0.92 in the right lobe among quality-passing examinations, and with similar performance across BMI strata. Liver stiffness measurement plays a central role across different liver disease etiologies, reflecting not only fibrosis but also dynamic changes related to inflammation and treatment response, as demonstrated in previous studies [21]. At the same time, acquisition feasibility emerged as a key practical limitation, particularly in the right lobe: only 42% of examinations met the IQR/Median < 30% criterion in the right lobe and 23% met the criteria in both lobes. Obesity did not decrease the EUS-pSWE feasibility. By contrast, VCTE showed the expected pattern for a transabdominal technique, with validity being numerically lower in obesity (93.5% vs. 98.6%). A plausible explanation for the comparatively low EUS-pSWE feasibility is that endoluminal acquisitions, especially from the duodenal bulb for right-lobe sampling, are more sensitive to subtle motion, suboptimal ROI orientation, and field artifacts, and the application of stringent IQR/Median thresholds may therefore exclude a substantial fraction of otherwise clinically interpretable traces in routine practice. An important distinction should be made between measurement-level reproducibility and run-level repeatability. The high ICC values reported in Table 3 reflect agreement within a single continuous acquisition, where two consecutive blocks of measurements are obtained under stable probe positioning. In contrast, the markedly lower ICCs observed in the run-level analysis (Table 4) compare summary measures (medians of five measurements) between consecutive blocks, effectively treating them as separate short acquisitions. This approach captures additional sources of short-term biological and technical variability, including subtle probe repositioning, respiratory fluctuations, and hemodynamic changes. Therefore, these findings should not be interpreted as being contradictory, but rather as reflecting different layers of variability that are inherent to EUS-pSWE measurements. Overall, EUS-pSWE demonstrates excellent intra-acquisition stability but may exhibit greater variability when acquisitions are considered as separate short sessions, particularly in obese individuals.

Diehl et al. reported that, although right-lobe EUS-SWE correlated strongly with histology, variance in paired measurements was 3.5-fold higher in the left lobe, effectively depressing reproducibility below the 0.90 threshold [22]. In contrast, the high ICC values observed in our cohort were obtained under routine clinical conditions, without protocol optimization or interim recalibration, indicating the stable short-term measurement reproducibility of EUS-SWE in this setting.

The diagnostic performance observed in our cohort closely aligns with the accuracy benchmarks reported in recent prospective studies. In a 274-patient series, AbiMansour et al. observed AUROCs of 0.73 (left) and 0.80 (right) for ≥F3 fibrosis and demonstrated a linear 0.11 kPa rise in magnetic-resonance elastography (MRE) for every 1 kPa increment on left-lobe EUS-SWE [23]. Our Pearson correlations with VCTE (r = 0.74 left; 0.71 right) are numerically identical, reinforcing that near-field measurements track parenchymal mechanics, rather than artefactual interface phenomena.

The pattern observed in obese participants—marked deterioration in single-measurement repeatability but preserved mean stiffness—aligns with findings from a dedicated MASLD study by Wang et al., in which EUS-SWE maintained an AUROC of 0.93 for advanced fibrosis in patients with a mean BMI above 40 kg/m^2^ and significantly outperformed VCTE across all fibrosis stages [24]. Bauer et al. tackled the same problem from the trans-cutaneous side: even with an enlarged deep-abdominal (DAX) probe, 20.9% of VCTE scans in biopsy-controlled obese subjects were either failed or unreliable, and diagnostic accuracy was rescued only when technically acceptable traces were achieved (ρ > 0.78) [25]. Taken together, these data support our observation that adiposity impairs transabdominal but not intraluminal elastography, positioning EUS-SWE as the preferred technique when BMI exceeds the operational limits of VCTE.

Inter-lobar behavior deserves emphasis. Although our limits of agreement (−2.8 to +3.2 kPa) were narrow, right-lobe acquisition via the duodenal bulb was technically more demanding and exhibited lower quality pass rates, whereas left-lobe measurements were more frequently feasible. These findings suggest that, although bilateral assessment remains ideal, single-lobe measurements may be acceptable in clinical practice when acquisition from both lobes is not feasible, provided that measurements are obtained under standardized conditions and are consistently from the same anatomical site. This is particularly relevant for longitudinal follow-up, where the reproducibility of acquisition conditions may be more important than absolute inter-lobar agreement. An important methodological aspect is that all EUS-pSWE acquisitions were performed under monitored propofol sedation, which may influence absolute liver stiffness values and contribute to the differences observed compared with transabdominal elastography performed in the awake state. In the pediatric literature, general anesthesia has been associated with significantly higher transient elastography (TE) values in healthy children (5.4 vs. 4.2 kPa; ≈+29%), and an individual patient data (IPD) meta-analysis identified sedation as an independent predictor of an increased liver stiffness measurement (LSM) [26].

Although adult data directly comparing “awake versus sedated” elastography remain limited, robust evidence indicates that LSM responds rapidly to hemodynamic changes and hepatic congestion (with dependence on central venous pressure), and that propofol sedation and endoscopic conditions (including insufflation) may alter abdomino-thoracic pressures and splanchnic circulation [27].

Therefore, absolute kPa thresholds obtained during EUS should be interpreted with caution. However, because all measurements in our study were performed under standardized sedation conditions, conclusions regarding intra-session reproducibility remain internally valid. Prospective studies directly comparing liver stiffness in the awake versus sedated state (ideally on the same day and within the same hepatic regions) are needed to quantify the magnitude and direction of this effect.

### 4.2. Study Limitations

First, liver biopsy was not performed; VCTE-derived stiffness served as a pragmatic reference but is imperfect, particularly in obesity. Misclassification could bias correlation estimates. Second, the single-center design limits generalizability to other platforms and operator cohorts. Third, only intra-observer—not inter-observer—reliability was assessed; multi-operator reproducibility remains to be established. Fourth, although our obese subgroup matched the real-world prevalence, extreme BMI > 40 kg m^−2^ was under-represented, tempering conclusions in morbid obesity. Finally, as all measurements were obtained within the same session, repeatability over longer intervals incorporating physiological variability warrants further investigation. EUS examinations were performed for various clinical indications, including pancreatic and biliary conditions, which may have influenced liver stiffness measurements. Although patients with overt biliary obstruction were excluded, other underlying conditions could still have contributed to the variability. The distribution of EUS indications was not systematically analyzed as a covariate, representing a potential source of residual confounding. Although learning curve effects have been highlighted as an important factor in elastography performance, this study was conducted using a single experienced operator, and no formal assessment of the operator learning curve or experience-related variability was performed. Another potential source of variability is the use of concomitant medications, particularly those prescribed in patients with metabolic dysfunction-associated steatotic liver disease. Such therapies may influence liver stiffness measurements through mechanisms not directly related to fibrosis. As medication use was not systematically recorded in our study, its potential impact on stiffness measurements cannot be excluded and may represent an additional source of residual confounding. 

## 5. Conclusions

Echoendoscopic pSWE demonstrates excellent intra-observer reproducibility under quality conditions, consistently exceeding the WFUMB threshold of 0.80 across all the analyzed subgroups. Obesity did not significantly affect the measurement reproducibility, although increased short-term variability between acquisition runs was observed, particularly in obese individuals.

While acquisition feasibility based on IQR/Median criteria was limited, all measurements fulfilled device-specific validity criteria (VSN >60%), suggesting that conventional quality thresholds may underestimate the practical applicability of EUS-pSWE. Moderate inter-lobar differences were observed, supporting the potential use of single-lobe assessment for longitudinal follow-up when acquisition conditions are standardized.

Overall, EUS-pSWE appears to be a technically robust and clinically promising modality for liver stiffness assessment, particularly in patients with limited transabdominal acoustic windows. Further multicenter studies are warranted to validate the quality criteria, assess interobserver reproducibility, and establish clinically relevant thresholds for disease stratification.

## Figures and Tables

**Table 1:** Baseline characteristics by BMI category.

Characteristic	Non-Obese (*n* = 74)	Obese (*n* = 46)	*p*
Age (years)	63.4 ± 19.7	59.2 ± 15.6	0.249
Male, *n* (%)	16 (30.2%)	27 (58.7%)	0.008
BMI (kg m^2^)	26.4 ± 4.0	30.6 ± 5.3	<0.001
Hypertension, *n* (%)	16 (39.0%)	29 (63.0%)	0.043
Diabetes, *n* (%)	3 (7.3%)	10 (21.7%)	0.075
ALT (U/L)	33.2 ± 18.1	43.4 ± 38.9	0.189

ALT, alanine-aminotransferase; BMI, body mass index; kg m^2^, kilograms per square meter; U/L, units per liter. Age and ALT compared with Welch’s *t*-test; categorical variables by χ^2^ test.

**Table 2:** Elastography performance and quality indicators by BMI category.

Metric	Non-Obese (*n* = 74)	Obese (*n* = 46)	*p*
Mean VCTE stiffness, kPa	6.27 ± 4.04	6.20 ± 4.30	0.929
Mean EUS-pSWE overall stiffness (10 measurements per lobe), kPa	9.05 ± 5.05	9.98 ± 6.50	0.411
Valid VCTE examination (IQR/Median < 30%), %	98.6%	93.5%	0.157
EUS-pSWE quality (IQR/Median < 30%), left lobe (%)	60.8%	50.0%	0.331
EUS-pSWE quality (IQR/Median < 30%), right lobe (%)	41.9%	41.3%	1
EUS-pSWE quality (IQR/Median < 30%), both lobes (%)	27.0%	17.4%	0.321
Left lobe stiffness, kPa	8.48 ± 4.74	9.18 ± 6.57	0.534
Right lobe stiffness, kPa	10.28 ± 6.28	12.37 ± 9.32	0.184

VCTE, vibration-controlled transient elastography; EUS-pSWE, endoscopic ultrasound point shear-wave elastography; IQR, interquartile range; and kPa, kilopascal. Continuous data by Welch’s *t*-test; proportions by χ^2^ test.

**Table 3:** Intra-observer reproducibility (ICC(3,1)) comparing consecutive measurement blocks (IQR/Median < 30%).

Group	*n* (Left Valid)	Left Lobe ICC(3,1)	*n* (Right Valid)	Right Lobe ICC(3,1)
Whole cohort	68	0.97 (0.95–0.98)	50	0.92 (0.86–0.95)
Non-obese	45	0.95 (0.91–0.97)	31	0.94 (0.88–0.97)
Obese	23	0.98 (0.96–0.99)	19	0.91 (0.78–0.96)

ICC, intraclass correlation coefficient. ICC computed with two-way random-effects absolute-agreement model.

**Table 4:** Repeat vs. initial run medians.

Group	*n* (Left Valid)	Left Bias (run2–run1), kPa	Left LoA (kPa)	Left Median |Δ| (kPa)	Left Median CoV	*n* (Right Valid)	Right Bias (run2–run1), kPa	Right LoA (kPa)	Right Median |Δ| (kPa)	Right Median CoV
Whole cohort	68	−0.32 ± 1.29	−2.85 to 2.22	0.71	6.30%	50	0.23 ± 3.19	−6.01 to 6.48	1.1	8.40%
Non-obese	45	−0.40 ± 1.31	−2.96 to 2.17	0.67	6.00%	31	−0.32 ± 2.15	−4.54 to 3.91	1.25	9.00%
Obese	23	−0.15 ± 1.27	−2.64 to 2.34	0.82	9.20%	19	1.14 ± 4.31	−7.31 to 9.58	0.88	6.60%

**Table 5:** Reliability of single-shot pairs vs. median-of-five shots per run.

Group	Left Lobe ICC Single-Shot	Left Lobe ICC Median-of-5 Shots	Right Lobe ICC Single-Shot	Right Lobe ICC Median-of-5 Shots
Non-obese	0.77 (0.61–0.86) (*n* = 45)	0.95 (0.91–0.97) (*n* = 45)	0.78 (0.59–0.89) (*n* = 31)	0.94 (0.88–0.97) (*n* = 31)
Obese	0.88 (0.73–0.95) (*n* = 23)	0.98 (0.96–0.99) (*n* = 23)	0.70 (0.38–0.87) (*n* = 19)	0.91 (0.78–0.96) (*n* = 19)

**Table 6:** Pearson correlation analysis between modalities and lobes.

Comparison	*n*	Pearson r	*p* Value
Overall: VCTE vs. EUS overall	120	0.81	<0.001
Non-obese: VCTE vs. EUS overall	74	0.84	<0.001
Obese: VCTE vs. EUS overall	46	0.8	<0.001
VCTE vs. Left lobe EUS	120	0.74	<0.001
VCTE vs. Right lobe EUS	120	0.73	<0.001
Left vs. Right lobe EUS	120	0.73	<0.001

**Table 7:** Determinants of absolute run-to-run difference (ΔkPa).

Predictor	β (SE)	*p* Value
Age (years)	0.032 (0.017)	0.066
BMI (kg/m^2^)	−0.045 (0.059)	0.443
Adjusted R^2^	0.029	

## Data Availability

The data presented in this study are available upon request from the corresponding author.

## Abbreviations

ALD	Alcohol-Related Liver Disease
ALT	Alanine Aminotransferase
AST	Aspartate Aminotransferase
AUROC	Area Under the Receiver Operating Characteristic Curve
BMI	Body Mass Index
CI	Confidence Interval
CLD	Chronic Liver Disease
CoV	Coefficient of Variation
EUS	Endoscopic Ultrasound
EUS-pSWE	Endoscopic Ultrasound Point Shear-Wave Elastography
FS	FibroScan
ICC	Intraclass Correlation Coefficient
IQR	Interquartile Range
LSM	Liver Stiffness Measurement
MASLD	Metabolic Dysfunction-Associated Steatotic Liver Disease
MRE	Magnetic Resonance Elastography
NITs	Non-Invasive Tests
OSF	Open Science Framework
ROI	Region of Interest
SD	Standard Deviation
TE	Transient Elastography
ULN	Upper Limit of Normal
VCTE	Vibration-Controlled Transient Elastography
VSN	Velocity Signal-to-Noise Ratio
WFUMB	World Federation for Ultrasound in Medicine and Biology
